# Abietane Diterpenoids Isolated from *Clerodendrum bracteatum* and Their Antioxidant and Cytotoxic Activities

**DOI:** 10.3390/molecules26164870

**Published:** 2021-08-11

**Authors:** Pingting Li, Lingling Li, Qin Zhu, Mingfeng Xu

**Affiliations:** College of Life and Environmental Sciences, Hangzhou Normal University, Hangzhou 311121, China; 2018210313055@stu.hznu.edu.cn (P.L.); 2018210313054@stu.hznu.edu.cn (L.L.)

**Keywords:** *Clerodendrum bracteatum*, abietane diterpene, antioxidant activity, cytotoxic activity

## Abstract

Two new abietane diterpenoids (**1,2**), along with five known diterpenoids (**3**–**7**), were first isolated and purified from the stems of *Clerodendrum bracteatum*. The structures of the new compounds were established by extensive analysis of mass spectrometric and 1-D, 2-D NMR spectroscopic data. Their antioxidant activities were determined on DPPH radical scavenging and ABTS. The in vitro cytotoxic activities of the compounds were evaluated against the HL-60 and A549 cell lines by the MTT method.

## 1. Introduction

The genus *Clerodendrum* is a diverse genus with about 580 species of small trees, shrubs, or occasionally perennial herbs, mostly in the tropics and subtropics of the world, including Africa and southern Asia. A few species are found in South America, northern Australia, and eastern Asia [1]. The whole plant has been used for the treatment of bleeding, rheumatism, hemorrhoids, and lung cancer. Previous phytochemical investigations on this genus resulted in the isolation of various types of compounds, including flavonoid compounds, phenylpropanoid glycosides, sesquiterpenoids, diterpenoids, triterpenoids, alkaloids, and so on, which exhibited a broad range of biological activities, such as antioxidant, antitumor, antibacterial, and anti-inflammatory [2,3].

**Figure 1:**
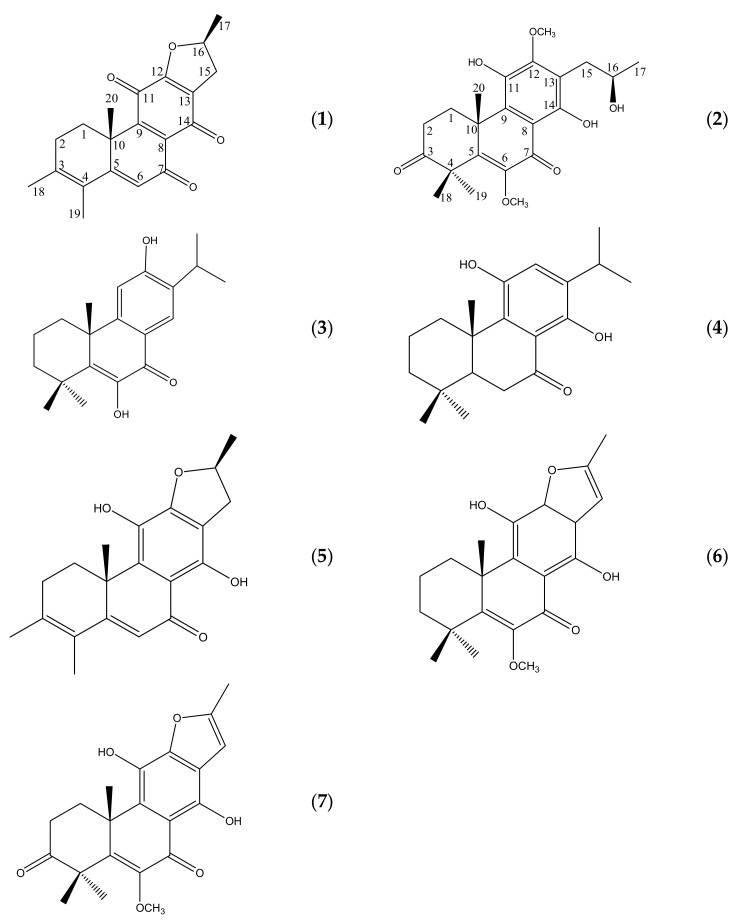
Chemical structures of compounds (**1**)–(**7**).

The chemistry of *Clerodendrum bracteatum* has been little investigated. As a part of ongoing research work on bioactive compounds, the stems of *C. bracteatum* were further investigated. This has led to the isolation and characterization of seven diterpenoids, including two new abietane diterpenoids (**1,2**), as well as five known diterpenoids (**3**–**7**) (Figure 1). Compounds **1**–**7** were evaluated for their cytotoxicity on two cancer cell lines, and their antioxidant activities were determined on DPPH radical scavenging and ABTS.

## 2. Results and Discussion

Compound **1,** ${\left[\alpha \right]}_{D}^{22}$: −0.5° (*c =* 0.1, CHCl_3_), was isolated as yellowish needles (acetone). The molecular formula of **1** was determined to be C_20_H_20_O_4_ (11 degrees of unsaturation) on the basis of its HRESIMS (*m/z* 323.1287 [M-H]^−^, calcd. for C_20_H_19_O_4_^−^, 323.1283). The UV spectrum of compound **1** showed absorption bands (229, 243, 268, 430 nm), which are characteristic of an aromatic moiety. The IR spectrum showed absorption peaks at 1690 cm^−1^ (carbonyl group) and 1675 and 1625 cm^−1^ (*p*-quinone moiety).

The ^1^H and ^13^C NMR spectra of **1** showed the presence of four methyls [δ_H_ 1.51 (3H, *d*, *J* = 6.2, H-17), 1.87 (3H, s, H-18), 1.88 (3H, s, H-19), 1.48 (3H, s, H-20)], three methylenes [δ_H_ 3.21 (1H, *d*, *J* = 15.2,10.1, H-15α), δ_H_ 2.68 (1H, *m*, H-15β), δ_H_ 1.58 (1H, *m*, H-1α), δ_H_ 2.74 (1H, *dd*, *J* = 16.5,7.6, H-1β), δ_H_ 2.22 (1H, *m*, H-2β)], δ_H_ 2.68 (1H, *m*, H-2β)], two methines [δ_H_ 6.37 (1H, *s*, H-6), 5.10(1H, *m*, H-16)]. The ^13^C NMR spectroscopic data of **1** displayed resonance for 20 carbons, which was confirmed by the DEPT and HSQC experiments to be 4 methyl groups, 3 methylenes, 2 methines, and 11 quaternary carbons. The three quaternary carbon signals at δ_C_ 183.9, 182.6, and 180.9 (C-7, C-11, and C-14, respectively) indicated the presence of three carbonyl groups, including a quinone moiety. Four downfield carbon signals at δ_C_ 130.6 (C-8), 153.3 (C-9), 157.6 (C-12), and 121.2 (C-13) were assignable to olefinic carbon atoms as members of the *p*-benzoquinone moiety, which was also supported by the germinal coupling constant of the C-15 methylene protons (*J* = 17.2 Hz). All these spectral data suggested the presence of an abietane diterpenoid. Our assignments were supported by HMBC data (See Supplementary Materials), which showed correlations from H-17 (δ_H_ 1.51) to C-15 (δ_C_ 34.3) and C-16 (δ_C_ 83.1), from H-15(δ_H_ 3.21 and 2.68) to C-12 (δ_C_ 157.6), and C-13 (δ_C_ 121.2). For biosynthetic considerations, Me-17 and Me-20 of compound **1** are expected to be β-oriented. The negative cotton effect at 307 nm in the CD spectrum indicated that the structure had the same abietane absolute configuration as mandarone A (Fan et al., 1999). All the above data established the structure of compound **1** as (10*S*,16*S*)-12,16-epoxy-17(15→16)-abeo-3,5,8,12,-abietatetraen-7,11,14-trione.

Compound **2**, ${\left[\alpha \right]}_{D}^{22}$: −18.1° (*c =* 0.1, CHCl_3_), was isolated as yellowish needle crystals (CHCl_3_). The molecular formula of **2** was determined to be C_2__2_H_28_O_7_ (9 degrees of unsaturation) on the basis of its HRESIMS (*m/z* 403.1761 [M-H]^−^, calcd. for C_2__2_H_27_O_7_^−^, 403.1757). The UV spectrum of compound **2** showed absorption bands (230, 280, 335 nm) that are characteristic of a benzene and a ketone. The IR spectrum showed absorption peaks at 1715 and 1650 cm^−1^ (two carbonyl signals) and 1620, 1610, and 1575 cm^−1^ (aromatic moiety).

The ^1^H and ^13^C-NMR spectra (Table 1) showed the presence of four methyls [δ_H_ 1.28 (3H, d, *J* = 6.1 Hz, H-17), δ_H_ 1.50 (3H, s, H-18), δ_H_ 1.56 (3H, s, H-19), and δ_H_ 1.45 (3H, s, H-20)], two pairs of doublet doublets at δ_H_ 1.85 (m, 1H), 2.71 (m, 1H), 2.73 (m, 1H), and 3.32 (m, 1H) corresponding to two methylene groups and one methine group at δ_H_ 4.17 (1H, m, H-16) together with two methoxyls [δ_H_ 3.88 and δ_H_ 3.85]. Additionally, strong chelation to a carbonyl at δ_H_ 13.35 (s) and one hydroxyl group at δ_H_ 5.85 (s) were also observed. Two ketone groups were observed at δ_C_ 213.7 (C-3) and δ_C_ 186.3 (C-7), and six aromatic C-atom signals at δ_C_ 115.5, 138.8, 131.9, 152.2, 119.0, and 155.5. All the above data, together with other spectroscopic characteristics, suggested that **2** was a diterpenoid [4].

The coupling system of the β-hydroxypropyl group [δ_H_ 2.86 (1H, overlapping, H-15α), 3.32 (1H, overlapping, H-15β), 4.17 (1H, m, H-16), 1.28 (3H, d, *J* = 6.1 Hz, H-17)] was determined based on its ^1^H-^1^H COSY spectrum, and this group was connected at C-13 based on HMBC correlations from the H-15 and 16 resonances with C-13 (δ_C_ 119.0),which suggested that the oxygenated substituent was placed at the C-16 position (–CH_2_CH(OH)CH_3_), and the side chain of 1 is not an isopropyl but rather a 2-hudroxy-*n*-propyl group (CH_3_-17 shifted to C-16 from C-15). The O-methyl proton resonance at δ_H_ 3.76 (3H, s) as well as the H-14, H-15α, and H-15β proton resonances exhibited long-range coupling with C-12 (δ_C_ 152.2) in the HMBC spectrum, which suggested the presence of a methoxy group at C-12. The ^1^H,^13^C long-range correlations between OCH_3_ (δ_H_ 3.88) and C-6 (δ_C_ 146.4) suggested the presence of a methoxy group at C-6. Therefore, compound **2** possesses an abeo-abietane diterpenoid framework with two OCH_3_ groups on C-6 and C-12. The absolute configuration of C-16 in the β-oxypropyl group was determined by a modified Mosher’s method, using C_5_D_5_N-*d*5 as the reagents. The treatment of **2** with (*R*)-(−)-MTPA and (*S*)-(+)-MTPA chlorides gave the 11,16-O-di-(*S*)-MTPA ester (a) and (*R*)-MTPA ester (b) of **2**, respectively. The value of the ^1^H -NMR differences [δ (ppm) = δa−δb] between the esters indicated that the absolute configuration of C-16 is *S*. Thus, the structure of **2** was elucidated as 11,14,16-trihydroxy-6,12-dimethoxy-17(15→16)-abeo-5,8,11,13- abietatetraen-3,7-dione (Figure 1).

The structures of the known compounds were established by comparison of their physicochemical and spectral data with reported data, and they were identified as 6,12-dihydroxyabieta-5,8,11,13-teraen-7-one (**2**) [5], 11,14-dihydroxy-8,11,13-abietatrien-7-one (**3**) [6], crolerodendrum A (**4**) [7], cyrtophyllone A (**5**) [8], and (10*R*,16*S*)-12,16-epoxy-11,14-dihydroxy-6-methoxy-17(15→16)-*abeo*-abieta-5,8,11,13-tetraen-3,7-dione (**6**) [9], respectively.

The antioxidant and cytotoxic activities of **1**–**7** were evaluated and are bsummarized in Table 2. The cytotoxic activities of the isolated compounds **1**–**7** were evaluated against two cell lines, and compounds **1** and **2** demonstrated cytotoxic activities against the HL-60 tumor (IC_50_ 21.22 ± 2.41 and 10.91 ± 1.62 μM) and A549 cell lines (IC_50_ 13.71 ± 1.51 and 18.42 ± 0.76 μM), respectively. Compound **2** also showed an IC_50_ value of 23.23 ± 2.10 and 15.67 ± 1.89 μg/mL for scavenging DPPH and ABTS^+^, respectively.

## 3. Materials and Methods

### 3.1. General Methods

Optical rotations were obtained using a Perkin–Elmer 241 automatic polarimeter (Perkin Elmer, Waltham, MA, USA), Absorption spectra were recorded by an ultraviolet-visible (UV-vis) light spectrophotometer (Lambda 35, PerkinElmer, Norwalk, CT, USA), Electronic Circular dichroism (CD) spectra were recorded on a Brighttime Chirascan spectrometer (Applied Photophysics Ltd., Leatherhead, UK); FTIR spectra were obtained by using a FTIR spectrometer (PerkinElmer, Norwalk, CT, USA); NMR spectra were taken on a Bruker AVANCE III 500 spectrometer (Bruker, Bremen, Germany); HRESIMS data were carried out on an Agilent 6210 ESI-TOF mass spectrometer (Agilent, Santa Clara, CA, USA); Silica gel (Qingdao Haiyang Chemical Group Co., Qingdao, China) and Sephadex LH-20 (Amersham Biosciences, Chicago, IL, USA) were used for column chromatography, Waters 1525 semi-preparative HPLC (Waters, MA, USA) coupled with a Waters 2996 photodiode array detector. A Kromasil C18 preparative HPLC column (250 mm × 10 mm, 5 μm) was used. Thin layer chromatographies (TLCs) (Merck, Darmstadt, Germany) were performed on silica-gel F_254_ plates and visualized under UV light, and by heating after spraying with 10% aq. H_2_SO_4_.

### 3.2. Plant Material

Woody branches and healthy stems of *C. bracteatum* were collected in July 2014 from the mountain of Dulongjiang, Yunnan Province, People’s Republic of China. The plant was identified by Dr. Chunhui Dai in Zhejiang Academy of Traditional Chinese Medicine. A voucher specimen (201418) has been deposited in the Key Laboratory for Genetic Improvement and Quality Control of Medical Plants of Zhejiang Province, Hangzhou Normal University.

### 3.3. Extraction and Isolation

Cut and air-dried stems (9 kg) of *C. bracteatum* were extracted under reflux with 90% ethanol (3 × 90 L) at 70 °C. The ethanol extracts were combined and evaporated to dryness under vacuum at 50 °C to afford a gummy residue (630 g). Part of the crude extract (500 g) was suspended in water (1 L) at 50 °C and fractionated with EtOAc (3 × 2 L) and n-BuOH (3 × 2 L) successively to yield the EtOAc (81 g) and n-BuOH (90 g) fractions, respectively.

The EtOAc extract (81 g) was fractionated by column chromatography on silica gel to give 19 fractions (F_1_–F_19_), eluted with petroleum ether–EtOAc mixtures of increasing polarity. Fraction F_11_, which eluted with petroleum ether–EtOAc (3:1), was chromatographed by reverse C18 silica gel column chromatography, eluting with MeOH in H_2_O with increasing polarity to give four subfractions (F_11__A_–F_11D_). F_11A_ (80 mg) was chromatographed over Sephadex LH-20 (CHCl_3_: MeOH, 1:1) to give **1** (16 mg). F_12_ (200 mg) was fractionated into three subfractions (F_12A_–F_12C_) through a Sephadex LH-20 column. F_12A_ was further purified by preparative HPLC (MeCN-H_2_O, 70:30, *v*/*v*) to yield compounds **3** (7.8 mg) and **4** (6.5 mg). Through similar procedures, F_12C_ yielded compounds **5** (12.0 mg) and **6** (9.9 mg). F_13_ (360 mg) was subjected to an MCI gel column eluted with MeOH-H_2_O (8:2, *v*/*v*) and further separated through Sephadex LH-20 (MeOH), and preparative HPLC (MeCN–H_2_O, 50:50, *v*/*v*) to give compound **7** (13.5 mg). Fr_14_ (90 mg) was purified by preparative HPLC (MeCN–H_2_O, 45:55, *v*/*v*) as the isocratic solvent system to obtain compounds **2** (11.5 mg).

#### 3.3.1. Compound **1**

Yellowish needles; ${\left[\alpha \right]}_{D}^{22}$: −10.5° (*c =* 0.1, CHCl_3_); UV (MeOH): 229 (2.77), 273 (2.63), 368 (2.13) nm. IR (KBr): νmax 3420, 2935, 2840, 1690, 1675, 1625, 1460, 1400, 1320, 1250, 1210, 1025 cm^−1^.^1^H and ^13^C-NMR spectral data (CDCl_3_, 500 and 125 MHz), see Table 1. HR-ESI-MS: *m/z* 323.1287 [M − H]^−^ (calcd. for C_20_H_19_O_4_, 323.1283).

#### 3.3.2. Compound **2**

Yellowish needle crystals; ${\left[\alpha \right]}_{D}^{22}$: −18.1° (*c =* 0.1, CHCl_3_); UV (MeOH): 230 (3.27), 258 (3.10), 280 (3.73), 353 (3.50) nm. IR (KBr): νmax 3430, 2950, 2875, 1715, 1650, 1620, 1465, 1430, 1380, 1360, 1285, 1025 cm^−1^. ^1^H and ^13^C-NMR spectral data (CDCl_3_, 500 and 125 MHz), see Table 1. HR-ESI-MS: *m*/*z* 403.1761 [M − H]^−^ (calcd. for C_22_H_27_O_7_, 403.1757).

### 3.4. Cytotoxicity Assay

The inhibitory effects of the compounds against HL-60 and A549 cells were determined using a MTT assay [10]. The cells (5000–10,000 per well) were cultivated in 96-well plates for 24 h. The medium was then replaced with new medium containing different concentrations of the compounds, and using cisplatin as a positive control. After incubation for 24 h, the medium was replaced by 100 μL of MTT, and the cells were further incubated for another 4 h at 37 °C to allow MTT formazan formation. Following incubation, the medium was replaced by acidic isopropanol (100 μL) to dissolve the formazan in each well. The absorbance was detected by a microplate reader (Multiskan Spectrum, Thermo Electron Corporation, Vantaa, Finland) at 570 nm. The concentration giving 50% inhibition (IC_50_) was calculated by NDST software, and each assay was performed in triplicate.

### 3.5. Free Radical Scavenging Assay and ABTS Test

The DPPH radical scavenging activity of the compound was determined according to the method of Ślusarczyk et al. with slight modifications [11]. Briefly, 0.2 mM solution of DPPH in methanol was prepared and 2.5 mL of this solution were added to 2.5 mL of compound solution in methanol at different concentrations. Then, 30 min later, the absorbance was measured at 517 nm in the UV spectrophotometry. A calibration curve was prepared using different Trolox concentrations (standard Trolox solutions ranging from 10 to 320 μM). The percentage inhibition activity was calculated as follows: (A_0_-A_t_)/A_0_ × 100%, where A_0_ is the absorbance of the control and A_t_ is the absorbance in the presence of samples.

The ABTS^+^ free radical scavenging assay was determined according to the method described by Wang with some modification [12]. ABTS+ radical cation was produced by mixing 7 mM ABTS^+^ solution with 2.45 mM potassium persulfate, and the mixture was stored at room temperature and in the dark for 12 h. Then, the ABTS^+^ solution was diluted with ethanol until its absorbance at 734 nm was 0.70. Next, 5 μL of sample solution was mixed with 2 mL of diluted ABTS^+^ radical solution and allowed to react for 6 min. The absorbance was measured at 734 nm by UV spectrophotometry. The scavenging activity was expressed as IC_50_ (the concentration of the tested sample required to scavenge 50% of ABTS), calculated by linear regression analysis. The experiment was conducted in triplicate.

### 3.6. Statistical Analysis

We described all values as the mean ± SD and analyzed by Graphpad Prism 6.0. To analyze the statistical significance among multiple groups, we used one-way analysis of variance (ANOVA) followed by Tukey post hoc test. *p*-values *<* 0.05 were considered to indicate statistical significance.

## 4. Conclusions

Two new (**1**,**2**) and five known abietane diterpenoids were isolated from *C. bracteatum*. These structures were identified by using spectroscopic methods. All the isolated compounds were evaluated for their cytotoxic and antioxidant activities. The results of the present study help to learn the potency of *C. bracteatum* as a potential source of natural antioxidants and suggests that *C. bracteatum*. might be explored as a viable source of potent antioxidants for the protection of food from oxidation. However, further research is needed to identify individual components that form an antioxidative system and develop their applications for food and pharmaceutical industries.

## Figures and Tables

**Table 1:** NMR spectroscopic data for compounds **1** and **2** in CDCl_3_ (*δ* in ppm, *J* in Hz).

NO	1 δ_H_	1 δ_C_	2 δ_H_	2 δ_C_
1	1.58, *m*, α 2.74, dd (16.5, 7.6)β	30.8, CH2	1.88, *m*,α 3.34, *m*,β	26.7, CH_2_
2	2.22, *m*, α 2.68, *m*, β	30.0, CH_2_	2.71–2.74, *m*, α 2.71–2.74, *m*, β	33.4, CH_2_
3		141.2, qC		213.7, qC
4		124.8, qC		49.5, qC
5		162.8, qC		156.7, qC
6	6.37, *s*	122.6, CH		146.4, qC
7		183.9, qC		188.3, qC
8		130.6, qC		111.5, qC
9		153.3, qC		138.8, qC
10		39.9, qC		41.7, qC
11		182.6, qC		131.9, qC
12		157.6, qC		152.2, qC
13		121.2, qC		119.0, qC
14		180.9, qC		155.5, qC
15	3.21, *dd* (17.2, 10.1) 2.68, *m*	34.3, CH_2_	2.91, *dd* (13.8, 4.0) 2.86, *dd* (13.7, 8.3)	33.0, CH_2_
16	5.10, *m*	83.1, CH	4.18, *m*	68.1, CH
17	1.51,d(6.3)	21.9, CH_3_	1.28, *d* (6.2)	23.9, CH_3_
18	1.87, *s*	14.8, CH_3_	1.50, *s*	25.9, CH_3_
19	1.88, *s*	20.9, CH_3_	1.56, *s*	22.6, CH_3_
20	1.48, *s*	24.2, CH_3_	1.45, *s*	20.1, CH_3_
6-OCH_3_			3.88, *s*	60.1, CH_3_
11-OCH_3_			3.85, *s*	61.9, CH_3_

**Table 2:** Antioxidant and cytotoxic activities of compounds **1**–**7**.

Compounds	DPPH	ABTS	Cytotoxity HL-60	Cytotoxity A549
**1**	42.34 ± 2.67	45.21 ± 3.79	21.22 ± 2.41	13.71 ± 1.51
**2**	23.23 ± 2.10	15.67 ± 1.89	10.91 ± 1.62	18.42 ± 0.76
**3**	45.63 ± 4.05	24.58 ± 2.55	39.54 ± 1.92	33.56 ± 2.51
**4**	48.23 ± 3.22	29.75 ± 2.56	26.88 ± 2.02	32.34 ± 3.04
**5**	125.65 ± 6.65	46.47 ± 3.88	65.12 ± 3.13	90.55 ± 6.22
**6**	118.42 ± 6.03	78.22 ± 6.13	67.55 ± 3.00	89.56 ± 5.28
**7**	72.59 ± 7.43	43.13 ± 1.01	43.12 ± 3.26	76.88 ± 5.10
**Trolox**	20.50 ± 2.22	13.69 ± 1.89	-	-
**Cisplatin**	-	-	11.70 ± 0.95	15.27 ± 1.00

Antioxidant and cytotoxic activities were expressed as IC_50_ (μg/mL) ± SD (*n* = 3) and IC_50_ (μM) ± SD (*n* = 3), respectively.

## Data Availability

Data are contained within the manuscript.

## Supplementary Materials

The following are available online. Figure S1: Chemical structure of compounds **1** and **2**, Figure S2: 1H NMR of compound **1**, Figure S3: 13C NMR of compound **1**, Figure S4: H-H COSY of compound **1**, Figure S5: HSQC of compound **1**, Figure S6: HMBC of compound **1**, Figure S7: 1H NMR of compound **2**, Figure S8: 13C NMR of compound **2**, Figure S9: 1H-1H COSY of compound **2**, Figure S10: HMBC of compound **2**, Figure S11: HSQC of compound **2**, Figure S12: ECD spectrum of compound **2**.

## Sample Availability

Not available.

## References

[1] Patel J.J., Acharya S.R., Acharya N.S. (2014). *Clerodendrum serratum* (L.) Moon.—A review on traditional uses, phytochemistry and pharmacological activities. J. Ethnopharmacol..

[2] Zhang S.L., Huang R.Z., Liao H.B., Wang H.S., Chen Z.F., Liang D. (2018). Cyclic pentapeptide type compounds from *Clerodendrum japonicum* (Thunb.) Sweet. Tetrahedron. Lett..

[3] Hu H.J., Zhou Y., Han Z.Z., Shi Y.H., Zhang S.S., Wang Z.T., Yang L. (2018). Abietane Diterpenoids from the Roots of *Clerodendrum trichotomum* and Their Nitric Oxide Inhibitory Activities. J. Nat. Prod..

[4] Xie W.D., Li X., Zhao J.H., Liu Y.H., Row K.H. (2012). Abietane diterpenoids from Isodon inflexus. Phytochemistry.

[5] Yang Z., Wang Q., Peng W., Zhan R., Chen Y. (2018). A new 12,17-cyclo-labdane diterpenoid from the twigs of Dacrycarpus imbricatus. Nat. Prod. Res..

[6] Hua L.P., Zhang Y.Q., Ye M., Xu W., Wang X.Y., Fu Y.H., Xu W. (2018). A new polyoxygenated abietane diterpenoid from the rattans of *Bauhinia championii* (Benth.) Benth. Nat. Prod. Res..

[7] María J.S.C., Maurizio B., María C.D.L.T., Franco P., Giuseppe S., Benjamín R. (1992). Rearranged abietane diterpenoids from the root of two Teucrium species.pdf. Phytochemistry.

[8] Liu Q., Hu H.J., Li P.F., Yang Y.B., Wu L.H., Chou G.X., Wang Z.T. (2014). Diterpenoids and phenylethanoid glycosides from the roots of *Clerodendrum bungei* and their inhibitory effects against angiotensin converting enzyme and alpha-glucosidase. Phytochemistry.

[9] Wang W.X., Xiong J., Tang Y., Zhu J.J., Li M., Zhao Y., Yang G.X., Xia G., Hu J.F. (2013). Rearranged abietane diterpenoids from the roots of *Clerodendrum trichotomum* and their cytotoxicities against human tumor cells. Phytochemistry.

[10] Tran T.H., Nguyen V.T., Le H.T., Nguyen H.M., Tran T.H., Do Thi T., Nguyen X.C., Ha M.T. (2021). Garcinoxanthones SV, new xanthone derivatives from the pericarps of *Garcinia mangostana* together with their cytotoxic and antioxidant activities. Fitoterapia.

[11] Ślusarczyk S., Cieślak A., Yanza Y.R., Szumacher-Strabel M., Varadyova Z., Stafiniak M., Wojnicz D., Matkowski A. (2021). Phytochemical Profile and Antioxidant Activities of Coleus amboinicus Lour. Cultivated in Indonesia and Poland. Molecules.

[12] Wang W., Gao Y.T., Wei J.W., Chen Y.F., Liu Q.L., Liu H.M. (2021). Optimization of Ultrasonic Cellulase-Assisted Extraction and Antioxidant Activity of Natural Polyphenols from Passion Fruit. Molecules.

