# Supplementary material for: Abietane Diterpenoids Isolated from Clerodendrum bracteatum and Their Antioxidant and Cytotoxic Activities
Source: Molecules. 2021 Aug 11;26(16):4870. doi: 10.3390/molecules26164870 (PMC8400359; doi:10.3390/molecules26164870)
Supplement: Supplementary file 1 [file molecules-26-04870-s001.zip › molecules-1242332-supplementary.pdf]

Supplementary Material

Article

Abietane Diterpenoids Isolated from *Clerodendrum Bracteatum* and Their Antioxidant and Cytotoxic Activities

Pingting Li, Lingling Li, Qin Zhu * and Mingfeng Xu *

College of Life and Environmental Sciences, Hangzhou Normal University, Hangzhou 311121, China; 2018210313055@stu.hznu.edu.cn (P.L.); 2018210313054@stu.hznu.edu.cn (L.L.)
* Correspondence: zhuqin@hznu.edu.cn (Q.Z.); zjxmf@163.com (M.X.); Tel.: +86-571-2886-1007 (M.X.)

Abstract: Two new abietane diterpenoids (**1**,**2**), along with five known diterpenoids (**3**–**7**), were first isolated and purified from the stems of *Clerodendrum bracteatum*. The structures of the new compounds were established by extensive analysis of mass spectrometric and 1-D, 2-D NMR spectroscopic data. Their antioxidant activities were determined on DPPH radical scavenging and ABTS. The in vitro cytotoxic activities of the compounds were evaluated against the HL-60 and A549 cell lines by the MTT method.

Keywords: *Clerodendrum bracteatum*; abietane diterpene; antioxidant activity; cytotoxic activity

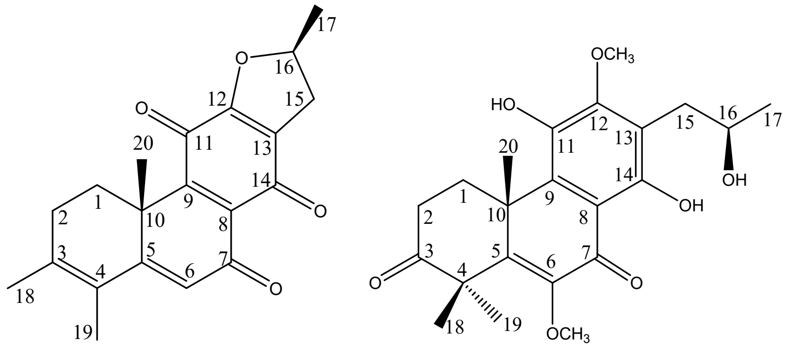

Figure S1. Chemical structure of compounds **1** and **2.**

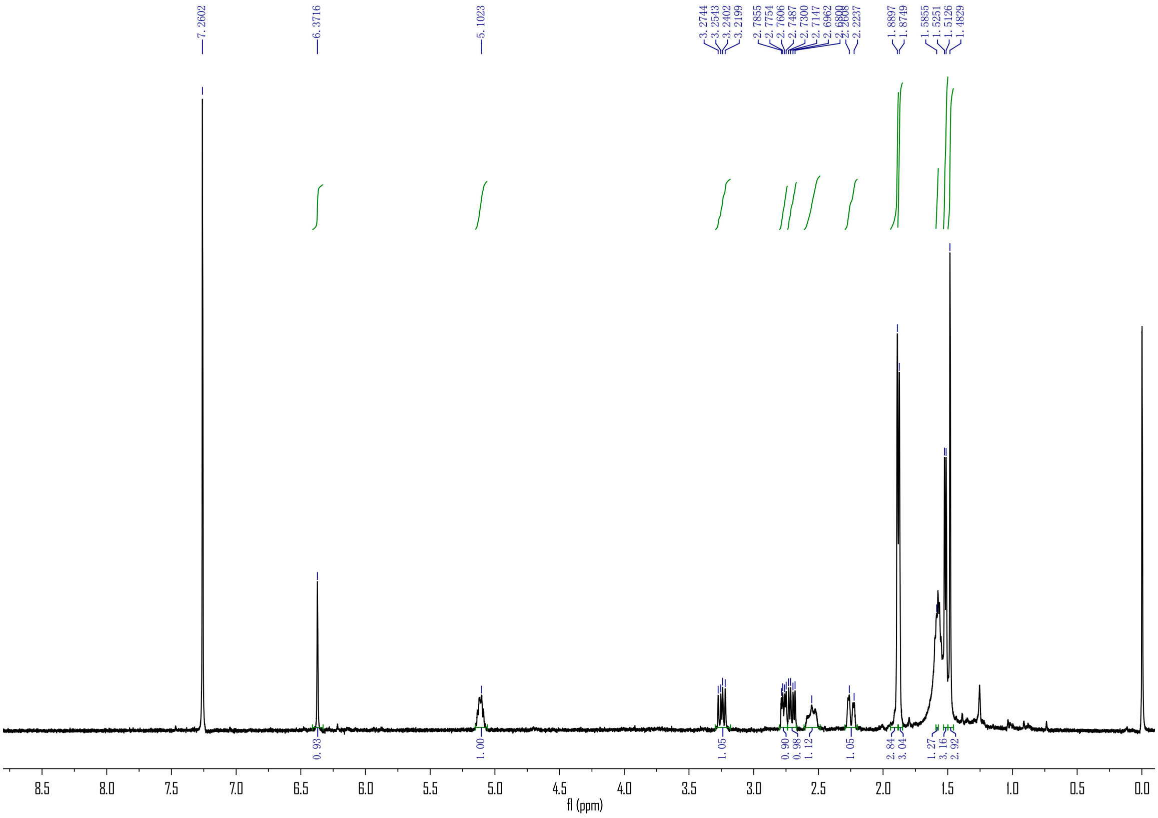

Figure S2. ^{1}H NMR of compound **1.**

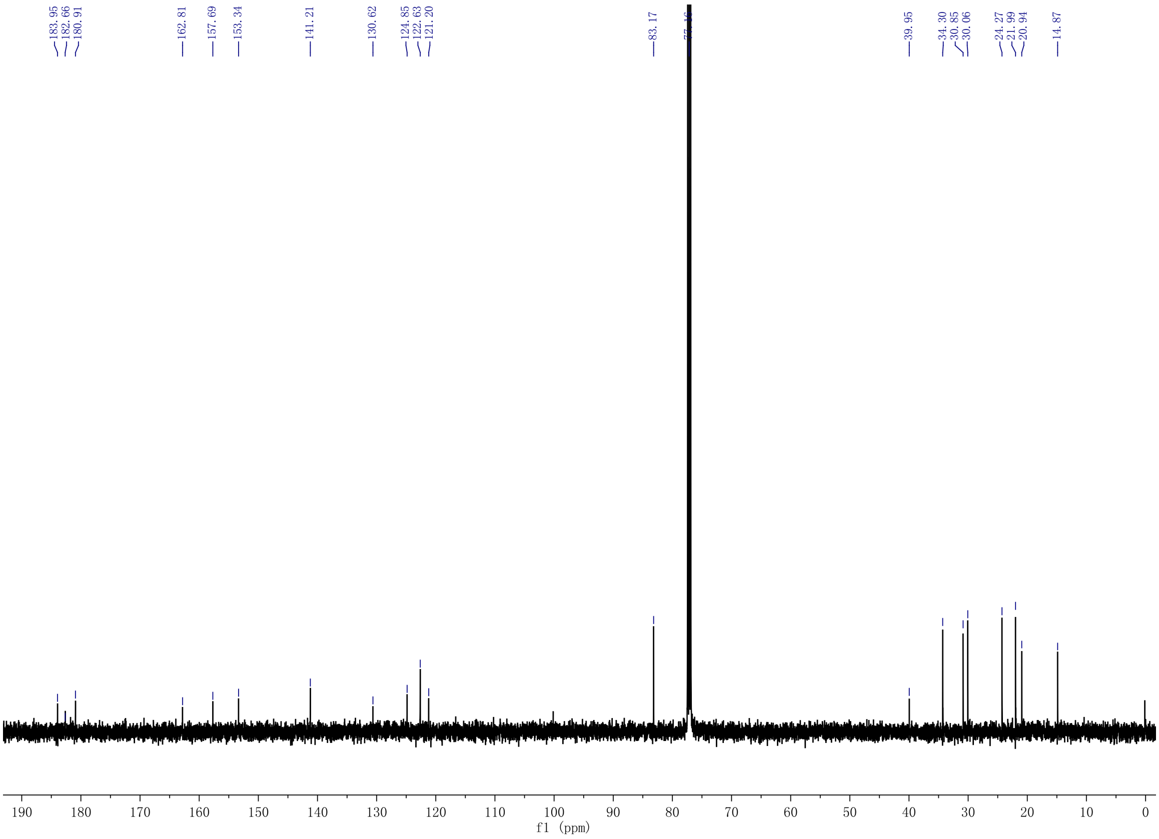

Figure S3. ^{13}C NMR of compound **1.**

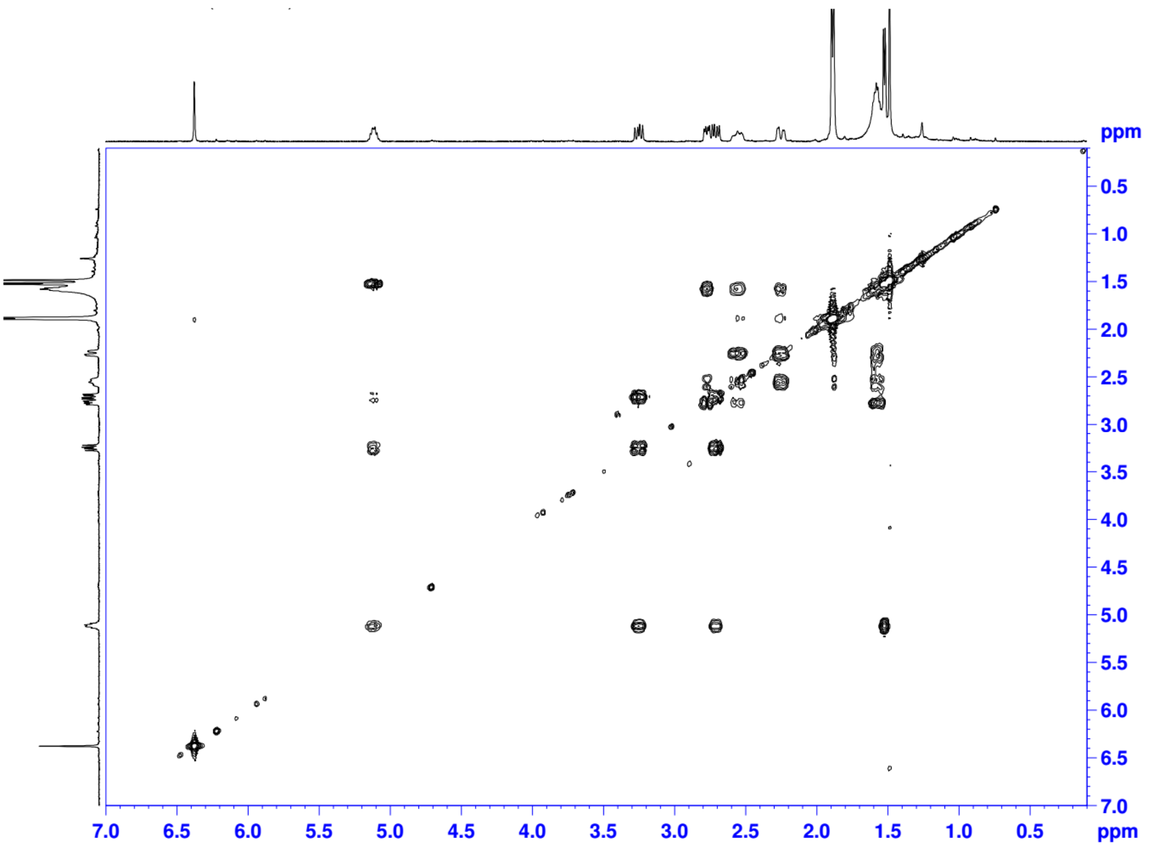

Figure S4. H-H COSY of compound **1.**

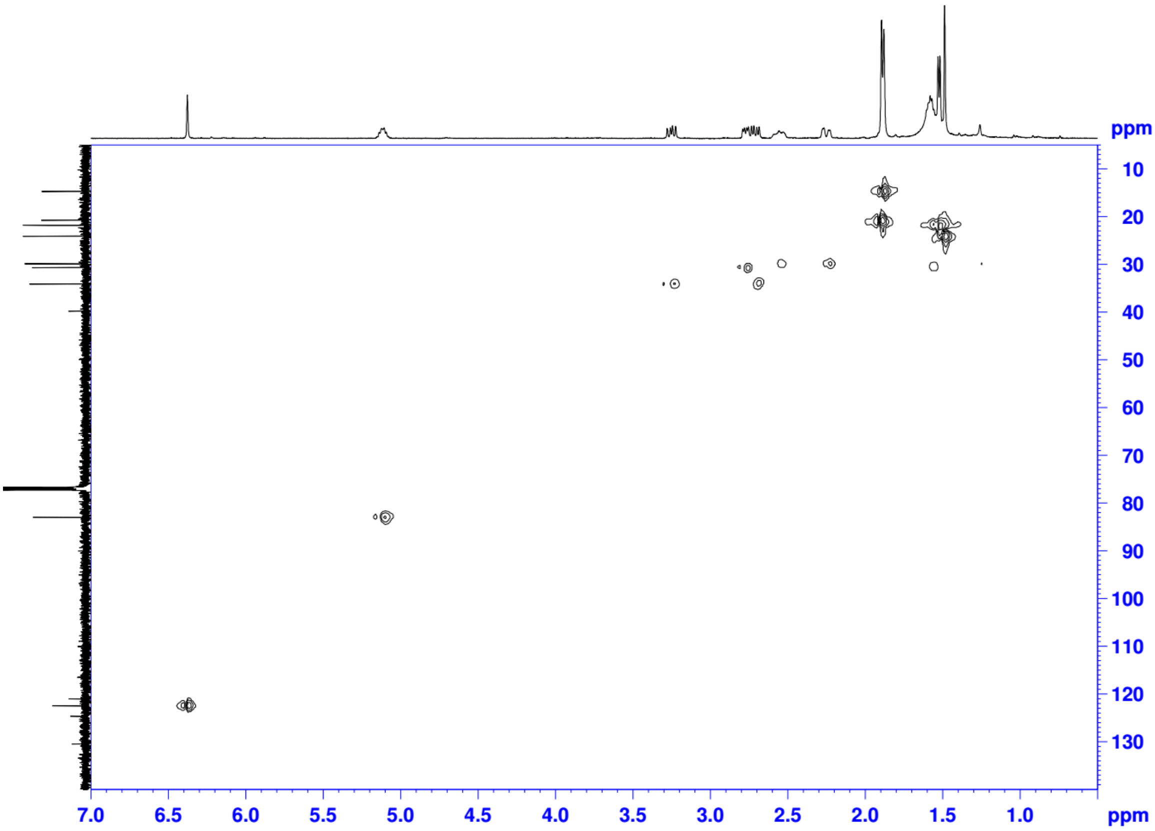

Figure S5. HSQC of compound **1.**

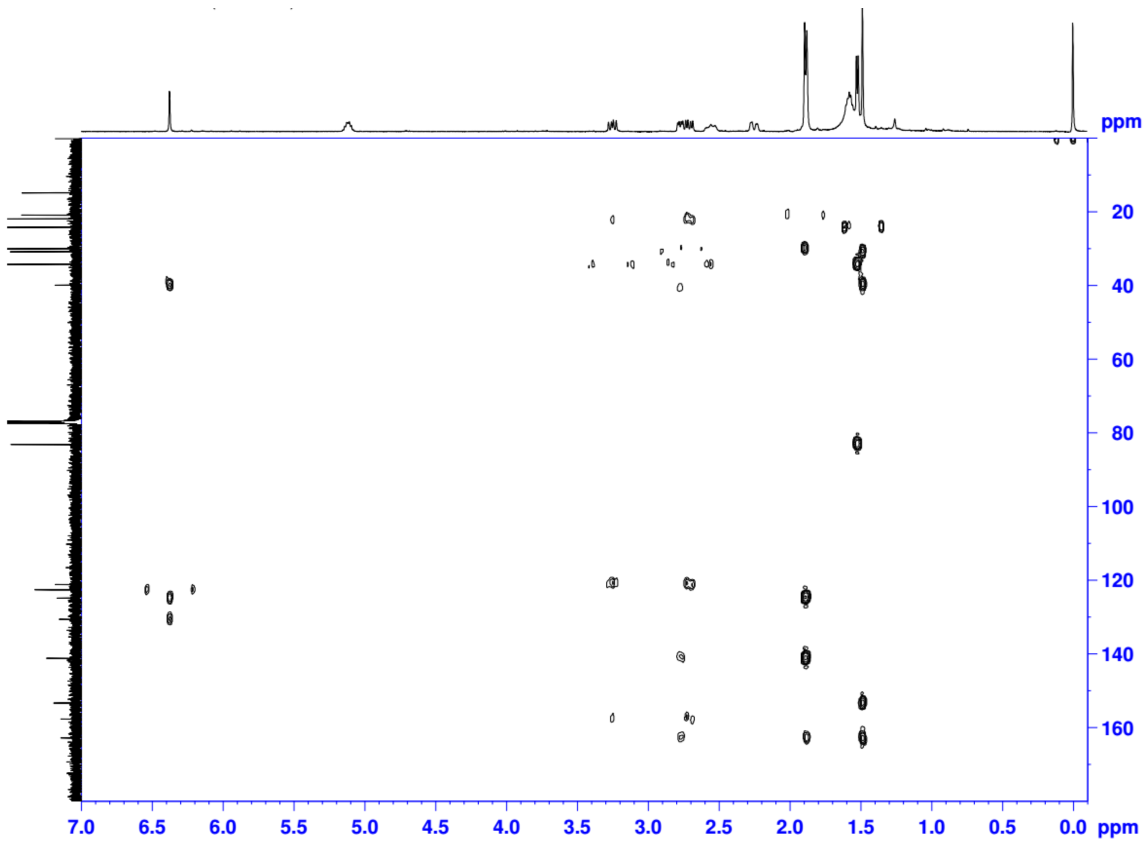

Figure S6. HMBC of compound **1.**

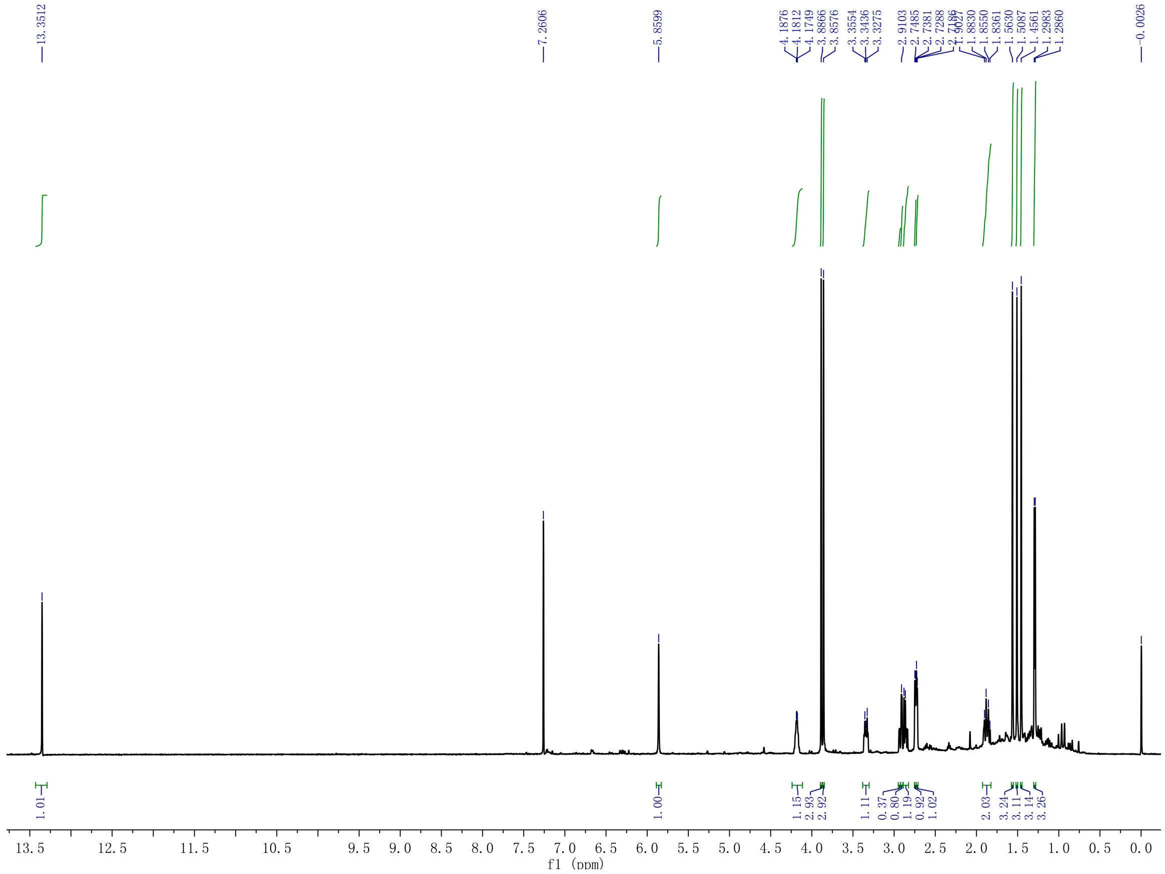

Figure S7. ^{1}H NMR of compound **2.**

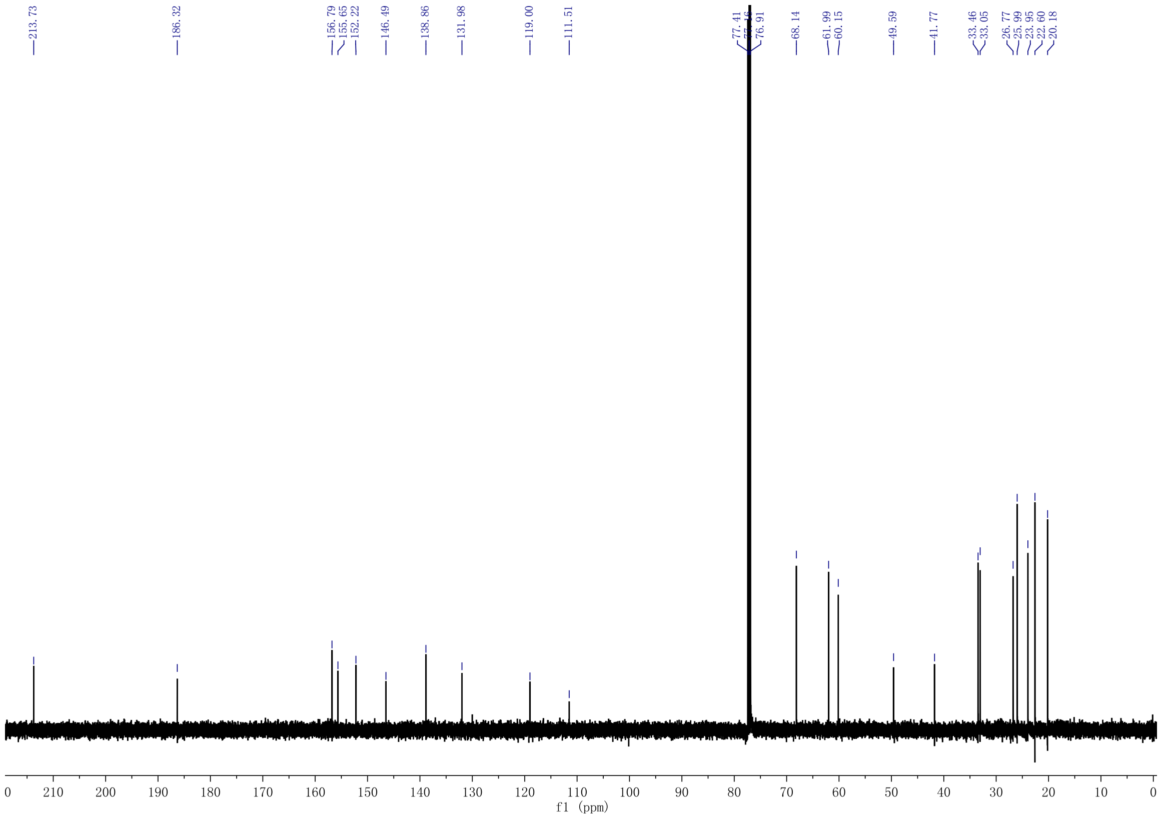

Figure S8. ^{13}C NMR of compound **2.**

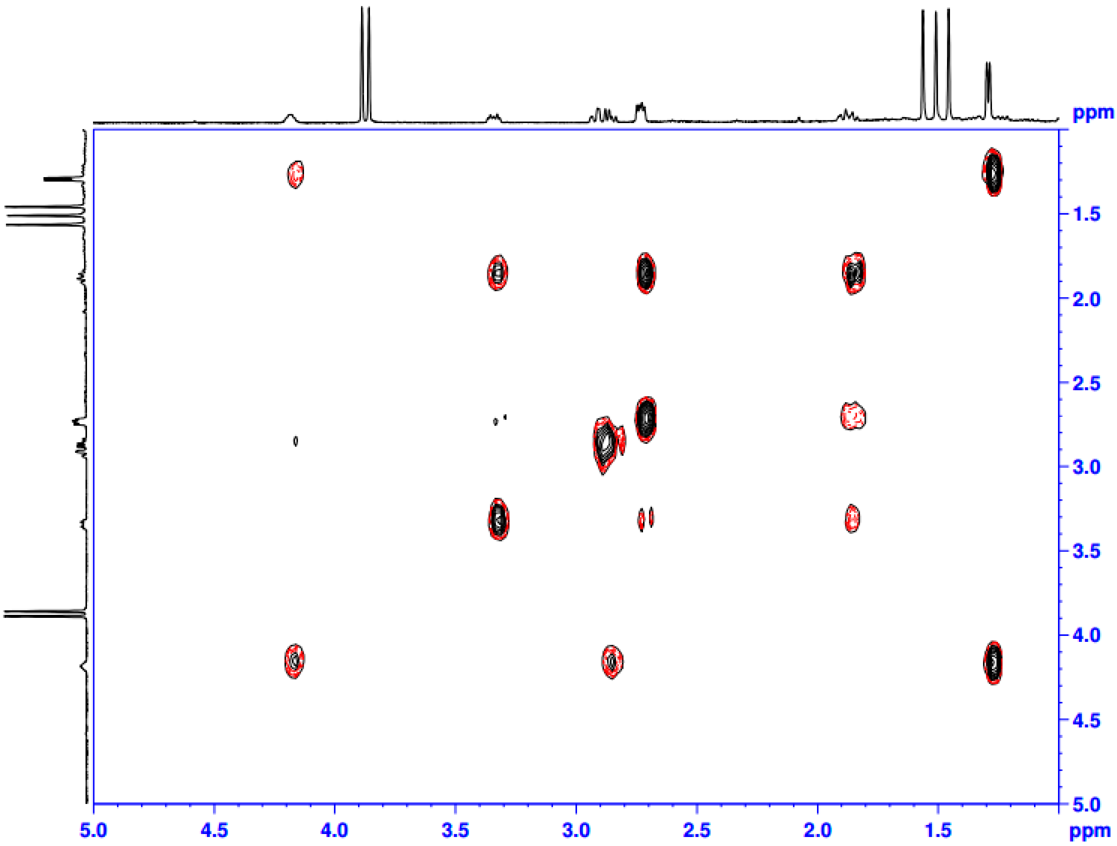

Figure S9. ^{1}H-^{1}H COSY of compound **2.**

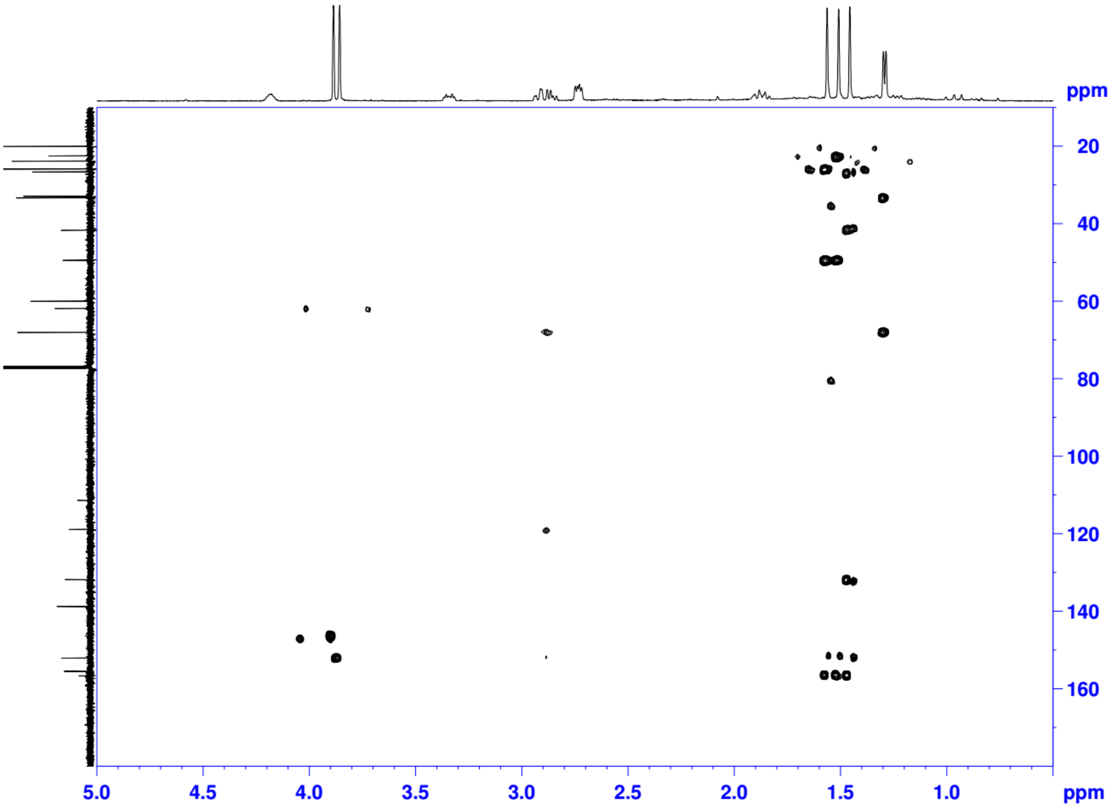

Figure S10. HMBC of compound **2.**

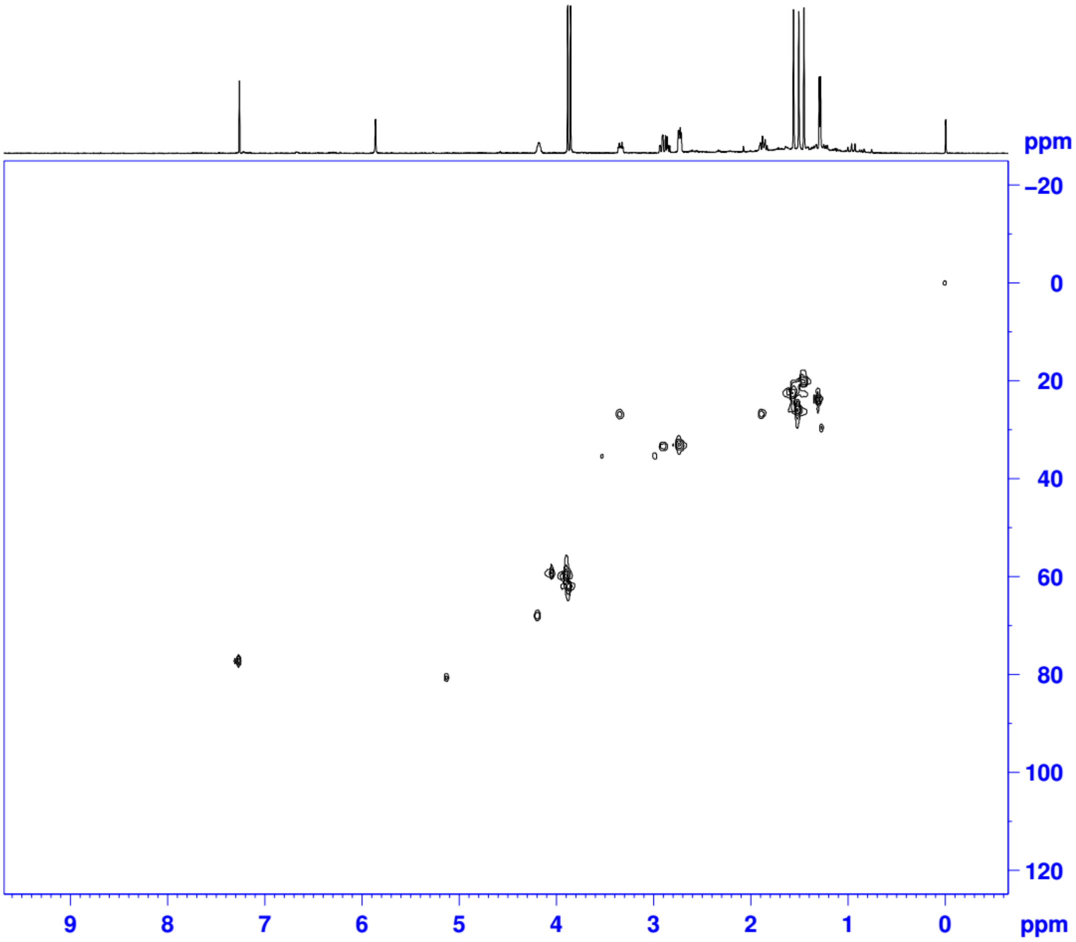

Figure S11. HSQC of compound **2.**

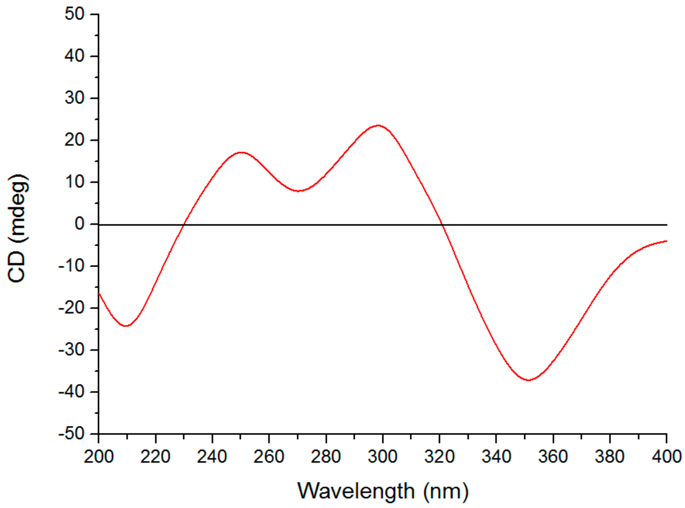

Figure S12. ECD spectrum of compound **2.**
